# Taxonomical and Phytochemical Characterisation of 10 *Stachys* Taxa Recorded in the Balkan Peninsula Flora: A Review

**DOI:** 10.3390/plants8020032

**Published:** 2019-01-29

**Authors:** Vjera Bilušić Vundać

**Affiliations:** University of Zadar, Department of Health Studies, Splitska 1, 23000 Zadar, Croatia; vjerab_2000@yahoo.com

**Keywords:** Lamiaceae, *Stachys*, essential oil, fatty acids, amino acids, flavonoids, iridoids, phytochemical, chemotaxonomy

## Abstract

The genus *Stachys* is one of the largest genera of the Lamiaceae, and it comprises about 300 species. Some species are highly polymorphic, with a number of infraspecific taxa. The aim of the present review is to summarise the available knowledge on 10 taxa belonging to the Balkan Peninsula flora (*S. alpina* L., *S. germanica* L., *S. menthifolia* Vis., *S. obliqua* Waldst. Et Kit., *S. officinalis* (L.) Trevis., *S. palustris* L., *S. recta* L. subsp. *recta*, *S. recta* L. subsp. *subcrenata* (Vis.) Briq., *S. salviifolia* Ten., and *S. sylvatica* L.) in order to enable insight into the identified biologically active substances and their possible application in intrageneric differentiation.

## 1. Introduction

*Stachys* L. (woundwort) comprises approximately 300 species, and is considered one of the largest genera of Lamiaceae, with nearly worldwide distribution. Most of these species occur in the warm temperate regions of the Mediterranean and southwest Asia, with secondary centres in North and South America and southern Africa; woundwort is not found in Australia and New Zealand [1]. The genus comprises 19 taxa in the flora of Croatia and neighbouring areas (Domac, 1973), among which eight species are endemic to the Balkan Peninsula or even narrower regions [2]. This article comprises phytochemical and taxonomical characterisation of 10 taxa: *S. alpina* L., *S. germanica* L., *S. menthifolia* Vis., *S. obliqua* Waldst. Et Kit., *S. officinalis* (L.) Trevis., *S. palustris* L., *S. recta* L. subsp. *recta*, *S. recta* L. subsp. *subcrenata* (Vis.) Briq., *S. salviifolia* Ten., and *S. sylvatica* L. among the presented species. Presented data also include chemotaxonomical guidance for highly polymorphic species of *S. recta,* with a proposal for the differentation of two of the presented subspecies [1,2,3,4,5,6,7]. *S. menthifolia* Vis. is not mentioned by Domac, but is listed in Flora Europaea as endemic species for Balkan Peninsula, and has been identified on several localities of Croatia and Montenegro [3,8,9]. The aim of present review is to find a scientific literature that contains research on the biologically active compounds of presented *Stachys* taxa belonging to the Balkan Peninsula flora and to report the findings on the chemotaxonomical composition that could help to differentiate within the *Stachys* spp. Presented data also include chemotaxonomical guidance for highly polymorphic species of *S. recta,* with a proposal for the differentation of two presented subspecies [1,2,3,4,5,6,7,10].

## 2. Taxonomy and Systematics

Genus *Stachys* is composed of annual or perennial herbs or subshrubs that are hispid or soft-pubescent. Leaves are sesille or stipulate, blade oblong to ovate, with serrate to crenate margins. Flowers are sessile or short-stalked, with two or more clustered in the axils of the leaves on the upper part of the stem. The calyx is bell-shaped, and has five lobes or teeth. Corolla has a narrow tube, generally with a short, pouched spur on the lower side of the tube. The upper lip is erect or generally parallel to tube axis, concave, entire (notched), and generally hairy, and the lower lip is perpendicular to the tube axis or reflexed, three (two)-lobed, and glabrous to hairy. The nutlets are oblong to ovoid [2,3,4,5,6,7,10].

**Figure 1:**
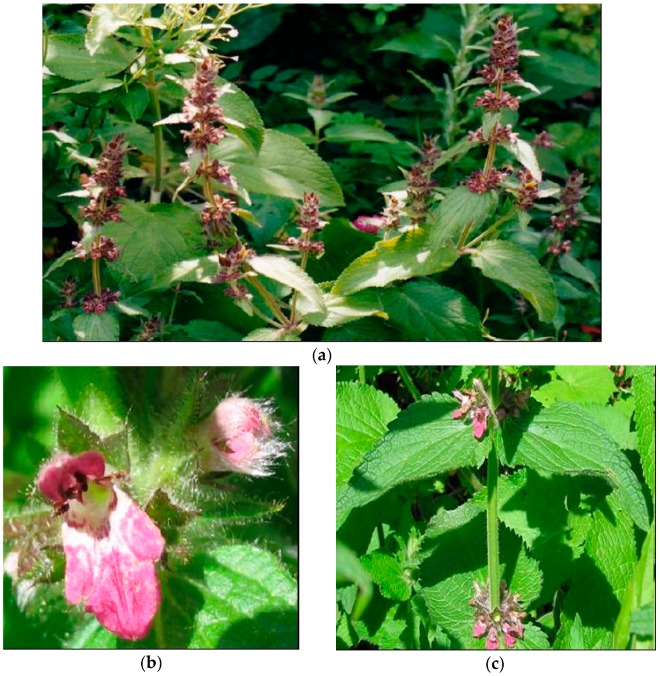
*Stachys alpina* L. (**a**) plant in flowering stage; (**b**) detail of the flower; (**c**) detail of leaf.

*S. alpina* L. (alpine woundwort) is a green, hirsute, glandular perrenial up to 100 cm in height. It grows in shadow on limestone soils from mountainous to subalpine areas [2,3,10] [Figure 1].

**Figure 2:**
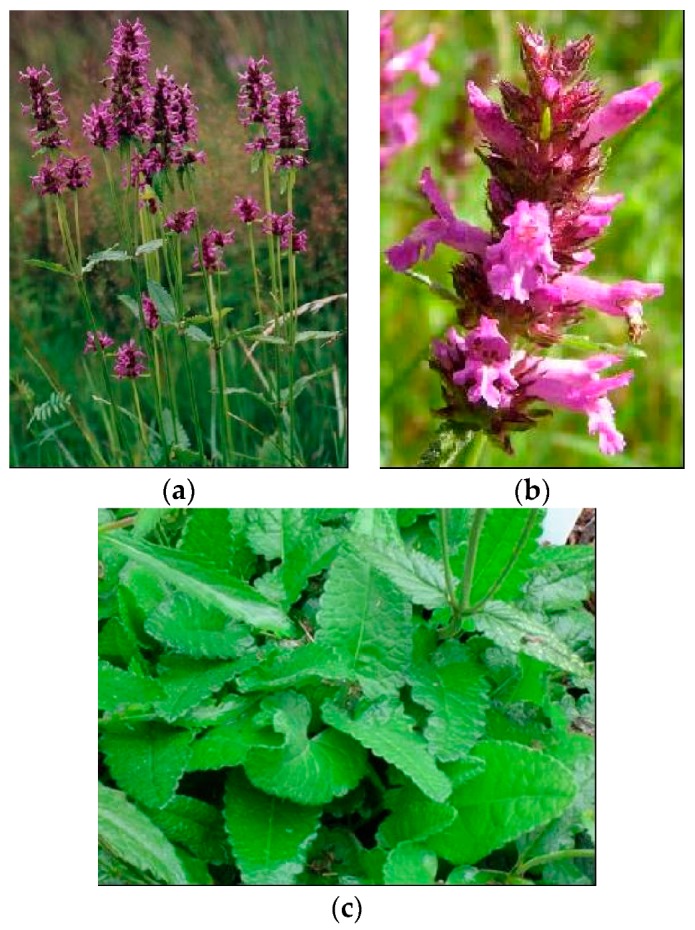
*S. officinalis* L. (**a**) plant in flowering stage; (**b**) detail of the flower; (**c**) detail of leaf.

*S. officinalis* L. (betony) is a perennial herb of 15–60 cm height, with sparse hairs, a short woody rhizome, well-marked basal rosettes, and erect, simple, or slightly branched stems. Betony occurs in open woods, stony grassy places, and dry meadows from the sea level up to 1600 m [2,3,10,11] [Figure 2].

**Figure 3:**
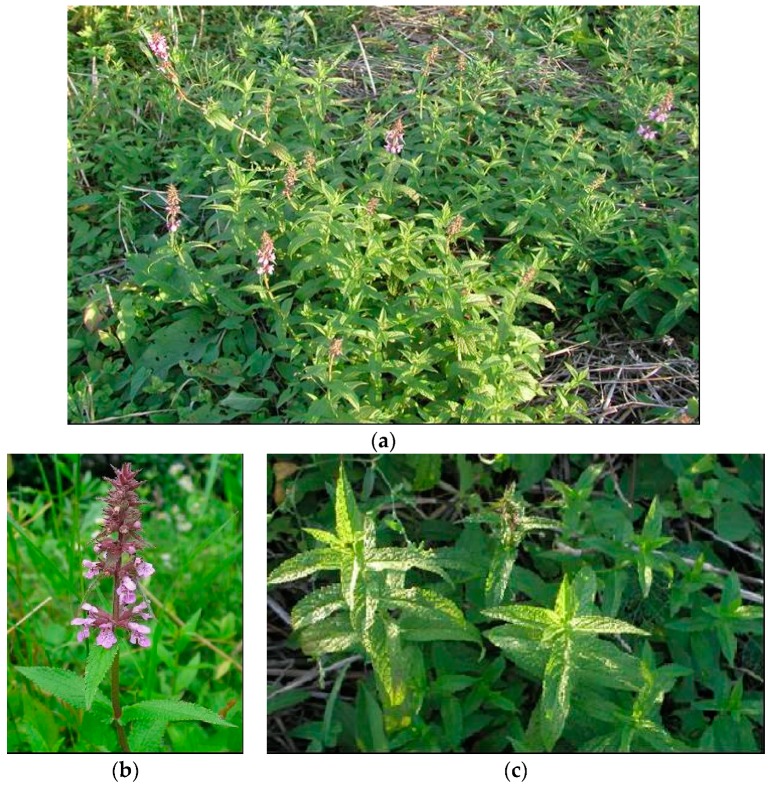
*S. palustris* L. (**a**) plant in flowering stage; (**b**) detail of the flower; (**c**) detail of leaf.

*S. palustris* L.(marsh woundwort) is a sparsely to densely hairy perennial plant of shady spots in marshes, bogs, ponds, lakes, and stream margins, damp places, roadside verges, and as a weed in cultivated fields growing up to 120 cm from a creeping rhizome [2,3,10] [Figure 3].

**Figure 4:**
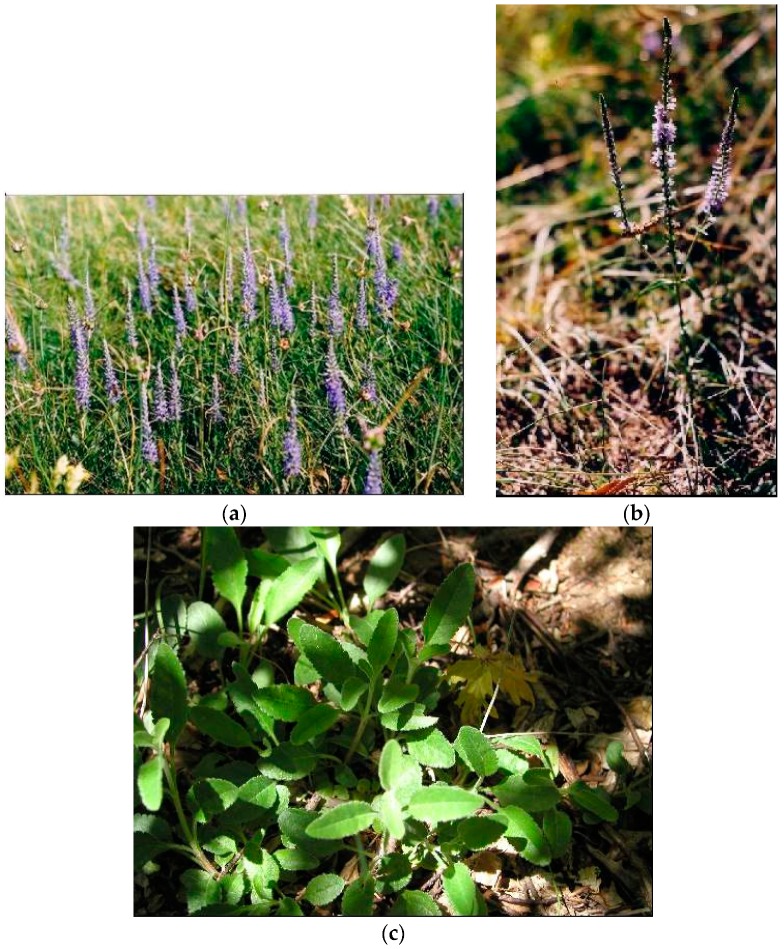
*S. recta* L. *subsp. recta* (**a**) plant in flowering stage; (**b**) detail of the flower; (**c**) detail of leaf.

*S. recta* L. subsp. *recta* (perennial yellow woundwort) is erect or ascending, subglabrous to sparsely hirsute, and usually an eglandular plant of up to 100 cm in height, which grows on different dry and stony habitats [2,3,10] [Figure 4].

**Figure 5:**
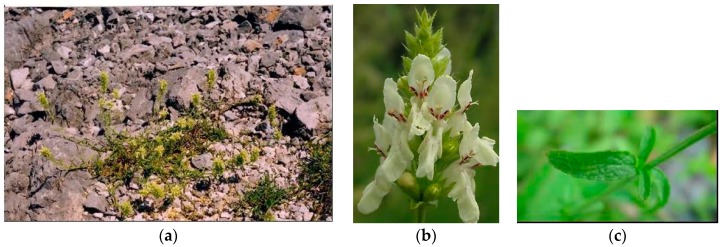
*S. recta* L. *subsp. subcrenata* (**a**) plant in flowering stage; (**b**) detail of the flower; (**c**) detail of leaf.

*S. recta* L. subsp. *subcrenata* (Vis.) Briq. has a very similar appearance and distribution as compared to the *S. recta* L. *subsp. Recta*, but has narrower leaves and often a glandular calyx with unequal teeth [2,3,10,11] [Figure 5].

**Figure 6:**
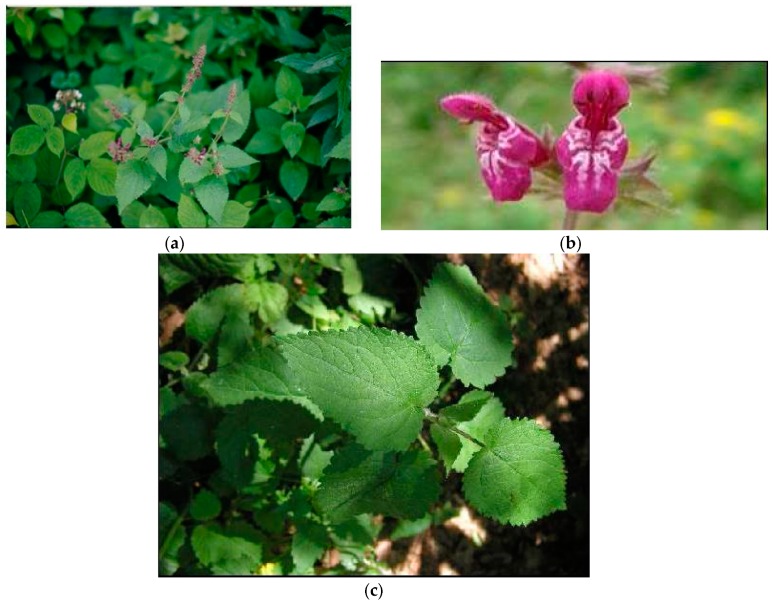
*S. sylvatica* L. (**a**) plant in flowering stage; (**b**) detail of the flower; (**c**) detail of leaf.

*S. sylvatica* L. (hedge woundwort) is an erect and hirsute perennial found in shady spots in woodland, forests, roadsides, alpine meadows, and grasslands, growing up to 120 cm. It differs from marsh woundwort in habitat preference, the broader leaves, and its characteristic, unpleasant smell when crushed, but the two can hybridise where they co-exist [2,3,10,11] [Figure 6].

**Figure 7:**
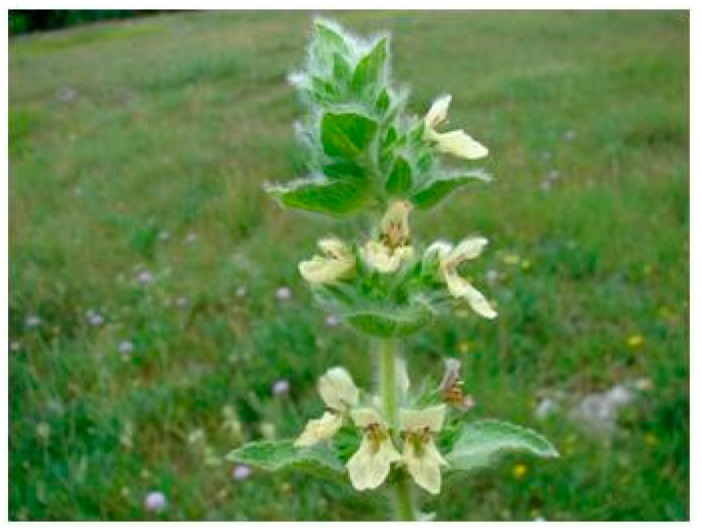
*Stachys obliqua* Waldst. et Kit. (by courtesy of Saxifraga Foundation).

*S. obliqua* Waldst. et Kit. is an erect lanate-hirsute perennial with oblong-lanceolate leaves that is native to the Balkan Peninsula [12] [Figure 7].

**Figure 8:**
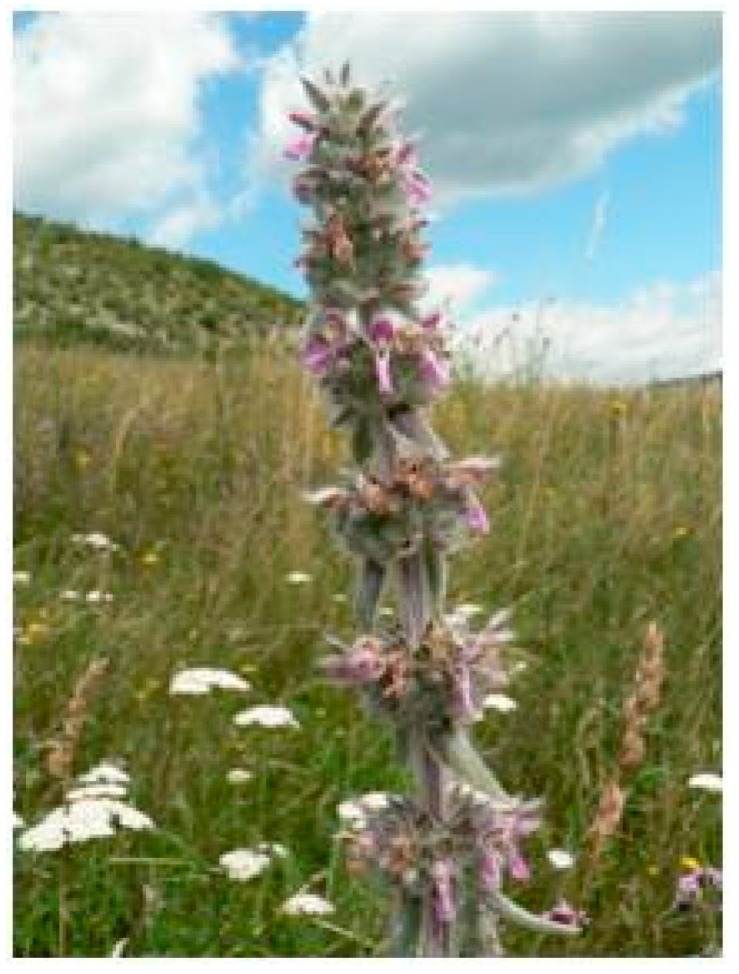
*Stachys germanica* L. (by courtesy of Saxifraga Foundation).

*S.germanica* L. (downy woundwort, german hedgenettle) is a densely tomentose and ascendent or erect biennial or perennial with well-marked basal rosettes, growing up to 120 cm and found in light forests, shrubby sunny slopes, rocks, and cut-over areas [11,12] [Figure 8].

**Figure 9:**
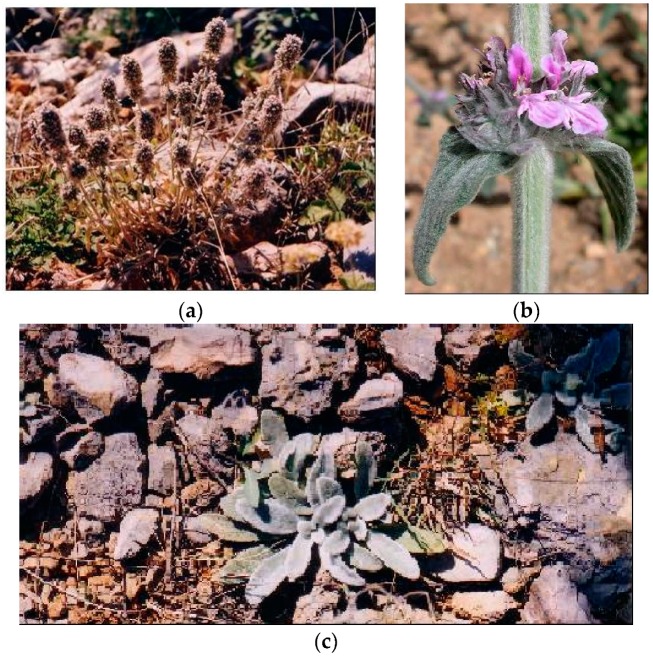
*S. salviifolia* Ten. (**a**) plant in flowering stage; (**b**) detail of the flower; (**c**) detail of leaf.

*S. salviifolia* Ten. (Mediterranean woundwort) is a densely tomentose or lanate-tomentose perennial growing up to about 80 cm that is widely distributed on rocky places in the littoral areas [2,3,10,11] [Figure 9].

*Stachys menthifolia* Vis. is an endemic species that was first described by Roberto Visiani (1829) at the locality of Kotor (Montenegro), who also identified this species in habitat near Dubrovnik (Croatia) [13]. According to the Flora Europaea, the species is endemic of the Balkan Peninsula, and spread in Albania, Greece, and Yugoslavia [3]. In 2002, Šilić and Šolić identified new localities in the eastern part of the Biokovo Massif [9]. *S. menthifolia* Vis. is a calciphilic plant, with the distinctive feature of a short, glandulary, and pilose stem, and is mostly present in the fissures of limestone or in the open limy rocky grounds, as in the Croatian localities. In the majority of the Montenegrin localities, the plants grow individually or in small groups, often in the strata of thermophilic woods and the underbrush of pubescent oak (*Quercetalia pubescentis*), in stone fissures, or in open surfaces, while the habitats of the new Croatian localities are mainly open limy rocky grounds with denser populations composed of larger individuals [9,14,15].

## 3. Phytochemical Characteristics

### 3.1. Essential Oils

Similar to many other representatives of the family *Lamiaceae*, *Stachys* species produce essential oil. Essential oils are complex mixtures and in spite of the large size of the genus *Stachys*, the composition of the volatile compounds is known in only a small number of species.

Several studies refer to the composition of the essential oil within the members of the present review: *S. alpina* [1,10], *S. officinalis* [1,16,17,18], *S. sylvatica* [1,16,17,19,20,21,22], *S. palustris* [1,23], *S. germanica* [16,17,24], S. *menthifolia* [14,15,25], *S.obliqua* [26,27,28], *S. recta* [1,17,25,29,30,31], and *S. salviifolia* [1,10].

Isocaryophyllene and b-caryophyllene, which are dominating constituents of the previously investigated essential oil from one population of *S. officinalis* in Montenegro [16], were totally absent in that from a population in Serbia, with germacrene D being the main compound [17]. Another study conducted in Serbia also confirmed sesquiterpene hydrocarbons as being the main fraction and identified germacrene D, *β*-caryophyllene, and *α*-humulene as the main constituents [18].

In a study conducted on samples from Croatia, germacrene D and (E)-caryophyllene were the main compounds of *S. officinalis*, with isocaryophyllene being totally absent [1].

Concerning the prior investigations of *S. sylvatica*, it is noteworthy that germacrene D is present in a large amount in the sample from Italy [19], and was also present in high proportions in the essential oil from Serbia [17], while the monoterpene fraction is present in all of the samples in a very low percentage only. *S. sylvatica* essential oil from Croatia was also rich in germacrene D, but in contrast to the oil from Italy and Serbia, monoterpene hydrocarbons were found in a large amount with α-pinene and β-pinene being the most abundant constituents of this fraction. The investigation of the chemical composition of essential oils from *S. sylvatica* collected from three different wild populations in Kosovo [20] showed that the leaves and flowers of *S. sylvatica* were characterised by three main constituents: α-pinene, β-pinene, and germacrene D, which is aligned with the findings on the main constituents that were identified in a study conducted in Croatia. Essential oil obtained from *S. sylvatica* sample collected in Turkey contained high proportions of germacrene D, α-pinene and β-caryophyllene, but the β-pinene constituent was not identified [21]. Another study conducted on a *S. sylvatica* sample collected in Turkey also implicated monoterpene hydrocarbons as the major constituents of essential oil, but with difference in a composition, with γ-muurolene, α-cedrene (11.2%), and limonene being the most abundant components [22].

Sesquiterpene hydrocarbons were the main group of essential oil constituents of *S. salviifolia* and *S. palustris* sample from Croatia, with germacrene D being the main constituent of *S. salviifolia*, in contrast to *S. palustris*, which contained a significant aldehyde fraction and a high amount of alcohols [1]. The essential oil from the aerial parts of *S. palustris* from both samples from Italy was characterised mainly by carbonylic compounds, fatty acids, and their esters, along with sesquiterpenoidic compounds and phenols [23,24].

The composition of the essential oil from one population of *S. recta* from Serbia [17] was similar to the oil from a population in Turkey [31] by the preponderance of 1-octen-3-ol. In contrast to the oil from one population in Greece [25] and to the oil from Turkey, in the oil from Serbia, linalool was present in very low percentage only. An analysis of *S. recta* oil from another population in Serbia revealed a different composition compared to other oils; germacrene D and E-caryophyllene were the main constituents of the essential oil, while 1-octen-3-ol was present in very low proportions, and linalool was totally absent [17]. The reason for these variations in the chemical composition of the essential oil was probably because there are different subspecies of *S. recta*, which can differ in their essential oil composition.

Two subspecies of *S. recta* (*S. recta* subsp. *recta* and *S. recta* subsp. *subcrenata*) that were analysed in the Croatian study were collected at the contiguous habitats. Although both subspecies had a high amount of sesquiterpene hydrocarbons, their composition differed; germacrene D was the main component of *S. recta* subsp. *subcrenata* essential oil, but its level is low in the *S. recta* subsp. *recta* oil. *S. recta* subsp. *subcrenata* was also characterised by a high percentage of oxygenated sesquiterpenes, whereas *S. recta* subsp. *recta* had a very low amount of these compounds. On the other hand, *S. recta* subsp. *recta* was rich in aldehydes, and had a rather high amount of ketones and fatty acids, which were present in the essential oil of *S. recta* subsp. *subcrenata* in a very low percentage only [1].

Stachynone and stachynene are constituents of the essential oil of *S. officinalis* and *S. recta*, as reported by Maly (1985), and were absent in all of the essential oils that were studied. One reason may be that these compounds decompose in the gas chromatographic injector [29].

Sesquiterpene hydrocarbons were the main group of constituents found in *S. menthifolia*, as demonstrated for several other *Stachys* species [25]. Abietatriene and 13-epi-manoyl oxide were present in high amounts in *S. menthifolia* from Greece [25], while samples collected in two different habitats in Croatia (Biokovo and Dubrovnik) differed in their essential oil composition. The major constituent found in the essential oil from the sample collected in Dubrovnik was, as in the sample from Greece, diterpenoid abietatriene (11.7%), with significant amounts of sesquiterpene hydrocarbons α-bisabolene (8.4%) and β-caryophyllene (7.4%). Oxygenated sesquiterpenes were the most abundant volatiles in the sample from Biokovo, followed by diterpene hydrocarbons. The content of sesquiterpene hydrocarbons was much higher in the sample from Dubrovnik, which also showed no presence of 4’-methoxyacetophenone. The authors attributed the differences in chemical oil composition to the different harvesting dates of the samples [14,15].

The *S. alpina* sample from Croatia showed the presence of high amounts of oxygenated sesquiterpenes, with a significant aldehyde fraction portion [1]. *S. germanica* essential oil samples from Serbia and Italy also showed oxygenated sesquiterpenes as the main constituents, with a sample from Serbia having a large amount of monoterpene hydrocarbons as well (11,3%) [17,24].

A similar composition was found for *S. obliqua* samples collected in Turkey, which had a significant portion of monoterpene hydrocarbons and a very high percentage of oxygenated sesquiterpenes with germacrene D as the main constituent [26,27,28].

A detailed overview of the main components (sesquiterpenes and monoterpenes) in the essential oil of *Stachys* taxa reviewed is presented in Table 1 and Table 2.

### 3.2. Other Biologically Active Substances

Similar to most of the plants belonging to the Lamiaceae family, the *Stachys* taxa have been submitted to several investigations in order to determine the content of the biologically active compounds. These investigations have reported the presence of flavonoids, phenolic acids, iridoids, and terpenoids.

Bilušić Vundać et al. investigated seven Croatian Stachys taxa (*S. alpina*, *S. officinalis*, *S. palustris*, *S. recta* subsp. *recta*, *S. recta* subsp. *subcrenata*, *S. salviifolia*, and *S. sylvatica*) in order to determine the content of the polyphenols, tannins, phenolic acids, and flavonoids, as well as the composition of flavonoids and phenolic acids. All of the taxa that were tested had a high content of total polyphenols, a medium content of total phenolic acids, and a rather low content of tannins and flavonoids. The most present phenolic compound in the investigated samples was found to be the chlorogenic acid (it was not present only in *S. recta* subsp. *recta*), and in samples of *S. recta* subsp. *recta* and *S. sylvatica*, the presence of isoquercitrin, luteolin-7-O-glucoside, and quercitrin were determined. Rutin was determined in samples of *S. recta* subsp. *recta*, *S. recta* subsp. *subcrenata*, *S. sylvatica*, and *S. salviifolia*. Although *S. recta* subsp. *recta* didn’t contain chlorogenic acid, it was found to be the richest in flavonoids whose presence was determined [32,33].

Karioti et al. (2010) determined 14 flavonoid glycosides in *S. recta*, with the majority of the constituents being the derivatives of isoscutellarein, and there being only four apigenin-*p*-coumaroyl derivatives [34].

Tundis et al. (2014) reviewed all of the available data on the biologically active substances in *Stachys* taxa, and found the following diterpenes to be present in the taxa that are included in this review: annuanone, stachylone, and stachone (*S. palustris*, *S. sylvatica*); abietal, abietatriene, stachysic acid, scareol, *trans*-Phytol, 6β-hydroxy-ent-kaur-16-ene, 6b,18-dihydroxy-*ent*-kaur-16-ene, kaur-16-ene,13-epi-manoyl oxide, and betolide (*S. sylvatica*); betonicoside A-D and *trans*-Phytol (*S. officinalis*); abietatriene, abieta-8, 11, 13-trien-7-one, 13-epi-manoyl oxide, *cis*-Phytol, and Labd-13-en-8,15-ol and kaur-16-ene (*S.menthifolia*); abietatriene and epi-Laurenene (*S. germanica*); and *cis*-Phytol, 11α,18-dihydroxy-*ent*-kaur-16-ene, kaur-16-ene, diacetyl derivatives, and monoacetyl derivatives of stachysolone (*S. recta*). Furthermore, in their overview of data on iridoids isolated in *Stachys* taxa, the following compounds were determined for the taxa presented: *S. recta* and *S. alpina* contained harpagide, ajugoside, aucubin, acetylharpagide, and harpagoside; *S. germanica* contained harpagide, aucubin, acetylharpagide, and harpagoside, *S. palustris* contained harpagide and aucubin; while *S. alpina* contained harpagide, ajugoside, aucubin, and harpagoside [35]. Furthermore, recent research studies of the active substances in *S. palustis* identified not only iridoids harpagide and 8-O-acetyl-harpagide, but also monomelittoside, which was the first identification of this compound in *S. palustris* species [36].

Bilušić Vundać (2006) identified several amino acids in *Stachys* species: aspartic acid (*S. alpina, S. sylvatica, S. officinals*, and *S. salviifolia*), asparagine (*S. sylvatica, S. alpina*, and *S. recta* subsp. *subcrenata*), γ-aminobutyric acid were determined in all of the investigated taxa (*S. alpina*, *S. officinalis*, *S. palustris*, *S. recta* subsp. *recta*, *S. recta* subsp. *subcrenata*, *S. salviifolia*, and *S. sylvatica*), as well as alanine, leucine, and serine [37].

## 4. Discussion and Conclusions

The presented studies revealed some differences between *Stachys* taxa, indicating the existence of a chemical polymorphism. The taxonomic relationships within the taxa are complex, and have been the objective of several investigations. Although different types of biologically active substances were determined within the investigated *Stachys* species, the main differentiation is based on essential oil and fatty acid composition. Tundis et al. (2014) focused on taxonomy within *Stachys* and *Betonica* subgenera, as well as infrageneric differentiation within *Stachys* species focusing on these chemotaxonomical markers. Based on the collected data on essential oil, three groups of *Stachys* species were observed: species mainly characterised by monoterpene hydrocarbons (to which *S. sylvatica* was assigned), species mainly characterised by oxygenated monterpenes (to which *S. recta* was assigned), and species that contain similar amounts of sesquiterpene hydrocarbons and oxygenated sesquiterpenes [35].

The essential oil approach, including its fatty acid composition, was taken by Bilušić Vundać (2006), who through principal components (PCA) and hierarchical cluster analyses (CA) differentiated seven *Stachys* taxa to two distinct groups, first comprising *S. alpina*, *S. recta* subsp. *Recta*, and *S. palustris*, and second comprising *S. officinalis*, *S. sylvatica*, *S. recta* subsp. *subcrenata*, and *S. salviifolia*. The species within the first group showed a high content of aldehyde components, and in the case of *S. alpina* and *S. palustris*, a high content of alcohol, while in the species within the second group, germacrene D was identified as the main component.

The essential oil and fatty acid approach was also used in chemotaxonomic differentiation by Kiliҫ at al. (2017), who defined germacrene D/β-caryophyllene the essential oil chemotype and 6-octadecanoic acid fatty acid chemotype for *S. sylvatica* [21].

The chemical investigations of *Stachys* species have determined the presence of monterpenes, sesquiterpenes, diterpenes, iridoids, phenylethanoids, flavonoids, and fatty acids as the main biological constituents. The content of these components and their occurrence in investigated species is variable, due to many external factors that impact the secondary metabolite composition of the plant species [1,35].

The differentiation of the presented *Stachys* species based on the available data on their chemical composition requests further chemotaxonomical and molecular research in order to present a key for infrageneric classification. Througout history, basic plant species and subspecies identification has relied mostly on morphological traits. For morphological characterisation, taxonomists have formulated a descriptor list, such as leaf shape and colour, flower colour, etc. With the development of scientific methods, chemotaxonomy research increased in order to enable separation within the taxa based on the presence and quantity of a specific chemical compound [38]. In recent studies, the authors have tried to resolve the classification of *Stachys* taxa based on the prevalent essential oil compositions from the aerial parts [10,21,26]. Nevertheless, the results vary, since different geographic localities, seasons, harvest periods, properties of soils, and climatic conditions strongly affect the secondary metabolite composition of plant species, especially their essential oil composition [1,21,35]. Therefore, traditional evaluation methods and chemotaxonomy should be combined with molecular markers that are not influenced by the environmental factors, for a better distinction among *Stachys* taxa.

Taking all of the above into consideration, a potential suggestion for differentiation can be suggested within the presented investigations only for two *S. recta* subspecies, which were collected at identical conditions and habitat (time of collection, harvest period, soil, and climate), which could support the opinion of Visiani (1829), who described *S. recta* subsp. *subcrenata* as separate species and named it *S. subcrenata* [10].

Although many constituents of this genus have been already identified, further investigations of *Stachys* spp. are required in order to clearly identify the chemotaxonomical markers for specific taxa and clarify their taxonomic relationships.

## Figures and Tables

**Table 1:** Main component groups found in *Stachys* taxa essential oil from aerial parts (monoterpene hydrocarbons: MH; oxygenated monoterpenes: OM; sesquiterpene hydrocarbons: SH; oxygenated sesquiterpenes: OS).

Taxa	Origin	MH	OM	SH	OS	References
*S. alpina*	Croatia	/	2.5	10.7	28.0	[1]
*S. germanica*	Serbia	12.3	0.8	37.8	4.8	[17]
	Italy, locality Parco Nazionale del Pollino	0.5	0.6	20.6	4.5	[24]
	Italy, locality Parco Nazionale delle Madonie	1.3	5.1	10.3	2.9	[24]
*S. menthifolia*	Croatia, locality Ploče	0.3	1.7	6.4	48.4	[14]
	Croatia, locality Dubrovnik	1.5	4.4	35.1	24.0	[14]
	Croatia, locality Biokovo	0.1–4.6	1.7–2.9	1.4–6.4	48.4–58.9	[14]
	Croatia, locality Vrgorac, alt. 180 *meter supra mare*	0.1	2.9	1.4	58.9	[14]
	Croatia, locality Vrgorac, alt. 30-130 *meter supra mare*	4.6	2.4	4.2	48.4	[14]
	Greece	4.3	0.5	15.3	14.2	[25]
*S.obliqua*	Turkey	10.3	/	72.2	4.1	[26]
*S. officinalis*	Croatia	2.4	0.3	70.4	18.7	[1]
	Serbia	0.3	0.4	71.1	7.7	[17]
	Serbia	0.8	3.7	69.1	14.8	[18]
*S. palustris*	Croatia	/	/	22.7	16.2	[1]
	Italy					[23]
	Italy	0.5	3.1	5.4	10.6	[24]
*S. recta*	Croatia (subs. *recta*)	4.1	2.9	22.2	2.1	[1]
	Croatia (subsp. *subcrenata*)	/	6.4	46.5	24.4	[1]
	Serbia	15.8	/	68.7	4.1	[17]
	Greece	1.6	41.9	6.6	/	[25]
	Serbia	2.2	/	9.3	4.6	[30]
	Turkey	17.7	29.7	13.9	/	[31]
*S. sylvatica*	Croatia	38.5	0.8	41.7	7.7	[1]
	Serbia	3.05	/	70.83	11.45	[17]
	Italy	1.7	/	72.6	6.6	[19]
	Turkey	48.9	1.2	32.1	2.6	[22]
*S. salviifolia*	Croatia	5.3	0.8	58.4	14.9	[1]

**Table 2:** Main sesquiterpenes and monoterpenes recorded in *Stachys* taxa (percentige above 5% presented).

Compound	Taxa	Origin	%	References
(E)-Caryophyllene	*S. officinalis*	Croatia	14.6	[1]
		Serbia	14.1	[18]
	*S. sylvatica*	Croatia	9.9	[1]
		Turkey	20.8	[21]
	*S. sylvatica (leaves)*	Italy	31.7	[19]
	*S. recta* subsp. *recta*	Croatia	5.4	[1]
	*S. menthifolia*	Croatia, locality Dubrovnik	7.4	[14]
	*S. officinalis*	Montenegro	22.9	[16]
Caryophyllene oxide	*S. palustris*	Croatia	16.2	[1]
		Italy	7.8	[23]
		Italy	7.8	[24]
	*S. menthifolia*	Croatia, locality Ploče	5.2	[14]
	*S. officinalis*	Montenegro	6.5	[16]
Germacrene D	*S. obliqua*	Croatia	20.1	[1]
	*S. officinalis*	Turkey	45.3	[26]
		Serbia	19.9	[18]
	*S. recta* subsp. *subcrenata*	Croatia	19.7	[1]
	*S. salviifolia*	Croatia	22.3	[1]
	*S. sylvatica*	Croatia	13.6	[1]
		Turkey	23.9	[21]
	*S. sylvatica (inflorescence)*	Italy	55.2	[19]
	*S. sylvatica (leaves)*	Italy	31.7	[19]
	*S. obliqua*	Turkey	8.2	[27]
		Turkey	6.2	[28]
α-Humulene	*S. officinalis*	Serbia	7.5	[18]
α-Pinene	*S. sylvatica*	Croatia	21.4	[1]
		Turkey	19.6	[21]
β-Pinene	*S. sylvatica*	Croatia	12.3	[1]
Linalool	*S.recta*	Turkey	13.0	[31]
(−)-β-Linalool	*S.recta*	Greece	33.9	[25]
(E)-Nerolidol	*S. alpina*	Croatia	12.3	[1]
γ-Cadinene	*S. recta* subsp. *recta*	Croatia	6.9	[1]
δ-Cadinene	*S. sylvatica (leaves)*	Italy	31.7	[19]
α-Cadinol	*S. recta* subsp. *subcrenata*	Croatia	9.5	[1]
β-Elemene	*S. salviifolia*	Croatia	9.4	[1]
α-Bisabolene	*S. menthifolia*	Croatia, locality Dubrovnik	7.4	[14]
Valeranone	*S. menthifolia*	Croatia, locality Vrgorac, alt. 30-130 *meter supra mare*	5.7	[14]
8-α-Acetoxyelemol	*S. menthifolia*	Croatia, locality Vrgorac, alt. 180 *meter supra mare*	21.3	[14]
		Croatia, locality Vrgorac, alt. 30-130 *meter supra mare*	6.9	
		Croatia, locality Ploče	12.8	
Limonene	*S. obliqua*	Turkey	6.2	[28]
	*S. sylvatica*	Turkey	37.0	[22]
Thymol	*S. obliqua*	Turkey	6.2	[28]
	*S. palustris*	Italy	5.8	[23]
α-Cedrene	*S. sylvatica*	Turkey	37.0	[22]
γ-Muurolene	*S. sylvatica*	Turkey	10.2	[22]
